# High prevalence of medicine-induced attempted suicides among females in Nuuk, Greenland, 2008–2009

**DOI:** 10.3402/ijch.v72i0.21687

**Published:** 2013-08-05

**Authors:** Lars Heymann Bloch, Gitte Hansen Drachmann, Michael Lynge Pedersen

**Affiliations:** 1 Dronning Ingrids Hospital, Nuuk, Greenland; 2 Primary Health Care in Nuuk, Nuuk, Greenland

**Keywords:** paracetamol, alcohol problems, sexual abuse, neglected children, psychosocial problems, vulnerability to emotional stress, emotional crises

## Abstract

**Background:**

The suicide rate in Greenland, especially among men, is among the highest in the world. Attempted suicide rates may be high also. However, the rates of attempted suicide are unknown.

**Objective:**

We aimed to estimate the age- and gender-specific incidence of attempted suicide using medicine in Nuuk in 2008–2009.

**Design:**

An observational retrospective study of consecutive medical records on patients admitted to Dronning Ingrids Hospital in Nuuk in 2008–2009 with possible medicine intoxication.

**Results:**

Seventy-four (60 females and 14 men) cases of attempted suicide using medicine were included. Of those, 43 used paracetamol alone or in combination with other medicine. The incidence of attempted suicide using medicine was higher among females than males (p<0.001). The highest incidence of attempted suicide with paracetamol was found among women aged 20–24 years (0.84%). The highest incidence of suicide with medication (1.31 per 100 inhabitants per year) was among women aged 15–19 years.

**Conclusions:**

The incidence of attempted suicide using medicine was high in Nuuk, Greenland, especially among women. The highest incidence of suicide attempts with medication was observed among women in the age group 15–19 years (1.31%). This may reflect psychosocial vulnerability among young people in Greenland. Initiatives to improve living conditions for children and adolescents are highly recommended to be initiated immediately.

Greenland is a modern society, with approximately 56,000 inhabitants distributed in 18 cities and a number of small human settlements (1). About one third of the population lives in Nuuk (1). Over the last 60 years, there have been some huge changes, where Greenland has gone from being a traditional Inuit hunting society to a modern society with the majority of the population living by wage income (1,2). In the wake of this rapid cultural and social change, there have been considerable changes in living conditions and health conditions (2–7).

Infectious diseases have declined markedly, while cardiovascular diseases, suicide and alcoholism have increased (5). Mental problems are thus increased in parallel with the societal development (6).

The incidence of suicide in Greenland is among the highest in the world (3,8). The highest incidence is found among men aged 15–24 years with annual instalments of 0.45–0.5% (3). The incidence is approximately four times higher for men than for women in Greenland (3). There are also regional differences within Greenland. The highest suicide rate is located in the capital Nuuk and in Eastern Greenland (9).

Not all suicide attempts result in death, which is true regarding medicine-induced attempts. It is possible that the incidence of medicine-induced suicide attempts is high among Greenlanders, but it is yet unknown. The purpose of this study is to estimate the prevalence of medicine-induced attempted suicides in Nuuk in the period 2008–2009 by review of all measured serum-paracetamol values and the medical record for all the patients who had their serum-paracetamol measured. Incidents are shown for both genders and a comparison is made with data from other Northern regions (women).

## Materials and methods

Patients admitted to Dronning Ingrids Hospital who were suspected of medicine-induced suicide attempts were reviewed between 2008 and 2009. The standard procedure for patients is to be brought to the emergency room and hospitalized from there. In the case of suspected medicine-induced attempted suicide, a serum-paracetamol (acetaminophen) is measured. This might not be the case for the minor hospitals on the coast. Due to geographic challenges and weather conditions, it is not always possible to get samples transported to Dronning Ingrids Hospital in Nuuk.

All patients in the period 2008–2009 who were tested for serum-paracetamol, were identified by an electronic search of the laboratory system on Dronning Ingrids Hospital.

An electronic query was made regarding every measured parcetamol value from 2008 to 2009. By reviewing medical records of the patients who tested positive for paracetamol, only those who resided in Nuuk were included in this study. Medical record information regarding residence, age, sex, alcohol, intoxication, reason for the intake of the medicine, and which type of medicine, were collected. Patients who stated that they had taken medicine as an attempted suicide, were recorded as cases with medicine-induced suicide attempts.

Patients who stated that paracetamol was among the medications ingested were recorded as cases with paracetamol-induced suicide attempts. Those who either consumed alcohol, or who were described as alcohol intoxicated in their medical record, were defined as having been drunk, despite serum-ethanol levels not being measured.

The age- and gender-specific incidence of medicine-induced suicide attempts was calculated in relation to the background population in Nuuk on 1 January 2009 (1). Ninety-five percentage confidence intervals (CI) were used in this study. Incidences were compared using a Chi-square test with a significance level of 0.05. We have been authorized by the Danish Authorities “Datatilsynet” to collect data. Our data have been anonymized, with identification of individuals no longer possible.

## Results

During the period 2008–2009, a total of 135 patients (96 women and 39 men) had their serum-paracetamol measured in the central laboratory in Nuuk. Of the 135 patients, 37 were excluded (23 women and 14 men), as they lived outside Nuuk. Of the remaining 98 patients, 24 patients were excluded (13 women and 11 men), as they had not taken medicine as a suicide attempt.

A total of 74 patients (14 men and 60 women) were counted as cases with medicine-induced suicide attempts. Of these, 43 patients (8 men and 35 women) had taken paracetamol as a part of the medication.

The prevalence of suicide attempts with medication was higher among women than among men (p<0.001). Alcohol intoxication was observed among 61% (45/74) of all cases of suicide attempts with medication.

**Fig. 1:**
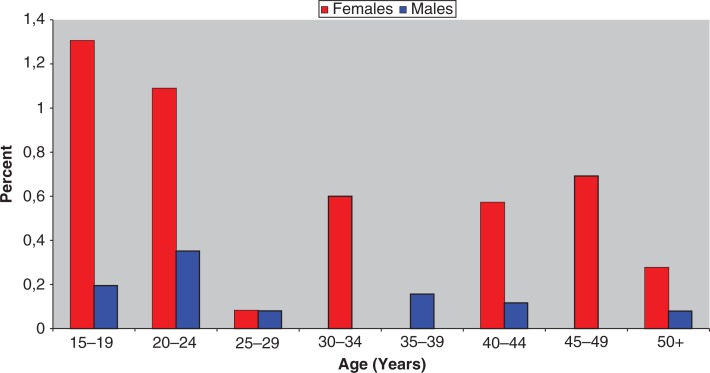
Incidence of medication-induced suicide attempts in Nuuk 2008–2009 by age group and gender.

The annual age- and sex-specific prevalence of suicide attempts with medication is shown in Fig. 1. The highest incidence was observed among women in the age group 15–19 years (1.31%).

There is an increase in cases of suicide attempts with medicine in women age groups 40–44 years and 45–49 years. The women older than 40 were in 89% (24/27) of cases assessed at admission to be alcohol intoxicated.

**Fig. 2:**
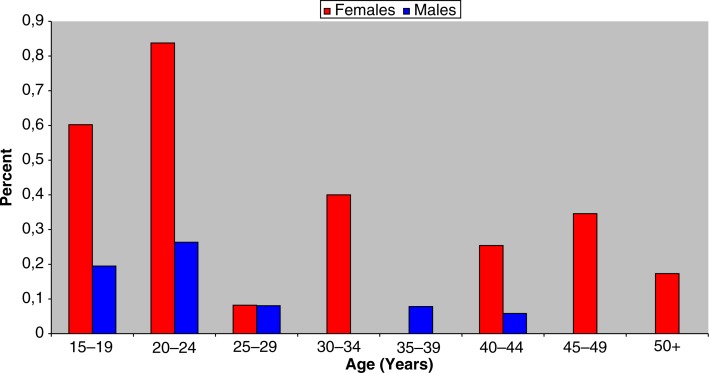
Incidence of paracetamol-induced suicide attempts in Nuuk 2008–2009 by age group and gender.

The annual age- and sex-specific prevalence of suicide attempts with paracetamol is shown in Fig. 2. The highest incidence is found among women aged 20–24 years (0.84%). Paracetamol was most frequently used by the 20–24 years age group. For this group, paracetamol was used in 77% (10/13) of suicide attempts with medication.

## Discussion

This study shows that suicide attempts with medication (Fig. 1) is very high in Nuuk, Greenland, especially among women. Prevalence of 1.31% in the women age group 15–19 years is the highest incidence described. Young Greenland men have the world record in suicides, nearly twice as high compared to other arctic countries (10).

The pattern, showing young women who attempts to commit suicide with medicine, is similar to the pattern in other countries such as England and Wales.

The very high prevalence of 1.31% of suicide attempts with medication in women aged 15–19 years (Fig. 1), as well as 0.60% in the same age group in which paracetamol has been among the medication (Fig. 2), is surprisingly high in comparison with England and Wales, where admission due to paracetamol poisoning in the 15- to 24-year-old women was 213.09 per 100,000 in 1995–2002 (0.21%). Figures from 2000 from Fyn in Denmark show an incidence of suicide attempts for young women in the age group 15–19 years, at 553 per 100,000, i.e. 0.55% (11). However, there is no information about how the young women have attempted suicide.

In Northern Quebec, Canada, a study of 99 Inuit between 14 and 25 years shows that 34% of these have previously attempted suicide. Thirteen percentage of these suicide attempts resulted in hospitalisation. Risk factors here are in the form of physical abuse and alcohol problems in the family (12).

Previous studies in Greenland show that 82% of those who have been exposed to frequent alcohol problems as well as sexual abuse have had serious suicidal thoughts (2,10). In relation to pure alcohol problems at home, the suicide thoughts will double in those cases where there has been sexual abuse (2,10). Correlation with education, profession, and socio-economic conditions was not significant (10). This indicates that in Greenlandic society, a significantly increased focus on the conditions for the children and young people is needed, especially in regard to alcohol problems at home and sexual abuse. Many appear to come out of the childhood and youth cases with significant mental health problems, which have emerged due to failure from caretakers.

A review of the medical files shows that in many cases, shortly before the suicide attempt, there were emotional crises caused by relationship issues, domestic quarrels, etc. This suggests general vulnerability to emotional stress, which is often seen among neglected children and young people (11).

The availability of over-the-counter drugs could also be a subject for improvement. Bjerregaard et al. looked at the incidence of admissions with paracetamol overdose in England and Wales, after the countries reduced the package size. The study showed a 24% reduction in paracetamol overdose admissions from 1997 to 2002 (13). Paracetamol is already sold in relatively small packet sizes in Greenland, so a reduction in packet size would probably not have a significant impact. However, there could be a requirement that over-the-counter drugs should be sold only by persons above 18 years, and that the seller must have a little knowledge about the medicine.

In our study, however, we did not focus on how the drug was acquired. In several cases, the medical records show that there were suicide attempts with combined intoxication with several different types of prescription medicine.

A weakness in our study should be mentioned. We have worked with small figures and in case of misinterpretation or randomness, it can give relatively large fluctuations on the results. However, a strength in the study is that all measured serum-paracetamol values in 2008 and 2009 are assessed together with the medical records. After exclusion of samples from the coast, a review of the medical records of the patients admitted from general health care was made.

## Conclusion

The presence of medicine-induced suicide attempts is very high in Greenland, especially among young women.

Comparing the incidence of suicide attempt with paracetamol in Nuuk with England and Wales shows that the incidents are significantly higher. For the group of 15–24-year-old women, the figures were 0.21% for England and Wales, compared with 0.60% for Nuuk women in the age group 15–19 years and 0.84% for the age group 20–24 years.

The same result arises when comparing the incidence of suicide attempt with medicine in Nuuk with Fyn, Denmark. In the age group 15–19 years, the incidents of suicide were 0.55% in Fyn and 1.31% in Nuuk.

This indicates significant psychosocial problems in this group of young women in Greenland. A fundamental approach to all facets of children and young people's lives, including family living, institutional and school relations, is required immediately.
